# Supplementary material for: Empirical methods for controlling false positives and estimating confidence in ChIP-Seq peaks
Source: BMC Bioinformatics. 2008 Dec 5;9:523. doi: 10.1186/1471-2105-9-523 (PMC2628906; doi:10.1186/1471-2105-9-523)
Supplement: Additional File 1 — A variety of html documents from the USeq web site detailing the available applications, their best usage, output file type descriptions, command line menus, etc. [file 1471-2105-9-523-S1.zip › USeqUserGuides/JavaDoc.zip!/JavaDoc/trans/graphics/VirtualCel.html]

VirtualCel


|  |  |  |
| --- | --- | --- |
| **Overview** \| **Package** \| **Class** \| **Use** \| **Tree** \| **Deprecated** \| **Index** \| **Help** | |  |
| **PREV CLASS**   **NEXT CLASS** | **FRAMES**    **NO FRAMES**     **All Classes** |  |
| SUMMARY: NESTED \| FIELD \| CONSTR \| METHOD | DETAIL: FIELD \| CONSTR \| METHOD |  |


---


## trans.graphics Class VirtualCel

```
java.lang.Object
  trans.graphics.VirtualCel
```

---

``` public class VirtualCel extends java.lang.Object ```

Builds virtual chips from float[][] files and saves each as a PNG image file.

---

**Constructor Summary**

`VirtualCel(float[][] intensityValues,
CelMasker celMasker)`
  
          For integration with CelMasker.

`VirtualCel(float[][] intensityValues,
java.io.File file,
int maxIntensityValueToPlot)`
  
          To write a matrix to png

`VirtualCel(java.lang.String[] args)`
  
          For generating png files.


| **Method Summary** | |
| --- | --- |
| `static void` | `main(java.lang.String[] args)` |
| `static void` | `printDocs()` |
| `void` | `processArgs(java.lang.String[] args)`             This method will process each argument and assign any new varibles |

**Methods inherited from class java.lang.Object**

`equals, getClass, hashCode, notify, notifyAll, toString, wait, wait, wait`

**Constructor Detail**

### VirtualCel

```
public VirtualCel(java.lang.String[] args)
```

:   For generating png files.

---


### VirtualCel

```
public VirtualCel(float[][] intensityValues,
                  CelMasker celMasker)
```

:   For integration with CelMasker.

---


### VirtualCel

```
public VirtualCel(float[][] intensityValues,
                  java.io.File file,
                  int maxIntensityValueToPlot)
```

:   To write a matrix to png


**Method Detail**

### main

```
public static void main(java.lang.String[] args)
```

---


### printDocs

```
public static void printDocs()
```

---


### processArgs

```
public void processArgs(java.lang.String[] args)
```

:   This method will process each argument and assign any new varibles


---


|  |  |  |
| --- | --- | --- |
| **Overview** \| **Package** \| **Class** \| **Use** \| **Tree** \| **Deprecated** \| **Index** \| **Help** | |  |
| **PREV CLASS**   **NEXT CLASS** | **FRAMES**    **NO FRAMES**     **All Classes** |  |
| SUMMARY: NESTED \| FIELD \| CONSTR \| METHOD | DETAIL: FIELD \| CONSTR \| METHOD |  |


---
